# What are the effective elements in patient-centered and multimorbidity care? A scoping review

**DOI:** 10.1186/s12913-018-3213-8

**Published:** 2018-06-14

**Authors:** Marie-Eve Poitras, Marie-Eve Maltais, Louisa Bestard-Denommé, Moira Stewart, Martin Fortin

**Affiliations:** 1 Département des sciences de la santé, Université du Québec à Chicoutimi, 555 Boulevard Université, Chicoutimi, Québec, G7H 2B1 Canada; 2 Département de médecine de famille, Université de Sherbrooke, Sherbrooke, Canada; 3 Centre for Studies in Family Medicine, The Western Centre for Public Health and Family Medicine, 2nd Floor, London, Canada

**Keywords:** Patient-centered-care intervention, Multimorbidity, Scoping review, Health-related positive outcomes

## Abstract

**Background:**

Interventions to improve patient-centered care for persons with multimorbidity are in constant growth. To date, the emphasis has been on two separate kinds of interventions, those based on a patient-centered care approach with persons with chronic disease and the other ones created specifically for persons with multimorbidity. Their effectiveness in primary healthcare is well documented. Currently, none of these interventions have synthesized a patient-centered care approach for care for multimorbidity. The objective of this project is to determine the particular elements of patient-centered interventions and interventions for persons with multimorbidity that are associated with positive health-related outcomes for patients.

**Method:**

A scoping review was conducted as the method supports the rapid mapping of the key concepts underpinning a research area and the main sources and types of evidence available. A five-stage approach was adopted: (1) identifying the research question; (2) identifying relevant studies; (3) selecting studies; (4) charting the data; and (5) collating, summarizing and reporting results. We searched for interventions for persons with multimorbidity or patient-centered care in primary care. Relevant studies were identified in four systematic reviews (Smith et al. (2012;2016), De Bruin et al. (2012), and Dwamena et al. (2012)). Inductive analysis was performed.

**Results:**

Four systematic reviews and 98 original studies were reviewed and analysed. Elements of interventions can be grouped into three main types and clustered into seven categories of interventions: 1) Supporting decision process and evidence-based practice; 2) Providing patient-centered approaches; 3) Supporting patient self-management; 4) Providing case/care management; 5) Enhancing interdisciplinary team approach; 6) Developing training for healthcare providers; and 7) Integrating information technology. Providing patient-oriented approaches, self-management support interventions and developing training for healthcare providers were the most frequent categories of interventions with the potential to result in positive impact for patients with chronic diseases.

**Conclusion:**

This scoping review provides evidence for the adaption of patient-centered interventions for patients with multimorbidity. Findings from this scoping review will inform the development of a toolkit to assist chronic disease prevention and management programs in reorienting patient care.

**Electronic supplementary material:**

The online version of this article (10.1186/s12913-018-3213-8) contains supplementary material, which is available to authorized users.

## Background

Patients with multimorbidity represent a significant portion of the primary healthcare population [1]. Although many definitions coexist in the literature, multimorbidity usually refers to the presence of multiple chronic or long-term conditions,that could include both physical and mental disease [2]. People affected by multimorbidity are using social and health care services more intensively compared to the rest of the population. As well, there is an economic burden associated with multiple chronic conditions [3], as multimorbidity has a large effect on patients’ daily life. More specifically, it reduces quality of life [4] and increases mental illness such as anxiety [5] and depression [6, 7]. Patients with multiple chronic diseases experience important treatment burden in terms of understanding and self managing their chronic conditions, attending multiple appointments, and managing complex drug therapies [8, 9]. In addition to a decrease in quality of life [10], multimorbidity increased care fragmentation, burden (care burden and treatment) and affected relationships with relatives [11–13]. This could lead to negative outcomes for patients and their caregivers [13].

For healthcare providers, managing patients with multiple chronic conditions represents a challenge given the complexity and the intensity of interventions [9, 14]. As an example, a minority of guidelines for patients with chronic disease consider multimorbidity as a norm in primary care settings. Most guidelines failed to discuss the burden of comprehensive treatment on patients or caregivers [15]. This does not reflect the reality of primary care where there is a very high prevalence of multimorbidity and is it difficult for healthcare providers to apply multiple recommandations to the same patient. This complexity calls for greater healthcare provider expertise [16], patient-centered care [17] and interprofessional collaboration [18]. Healthcare services have to be structured differently to reflect this intensity and integration of the interventions needed for complex patients [19].

In most cases, suitable interventions for patients with multimorbidity are multifaceted because they have to address a variety of individual needs [20]. Despite the specific needs of individual patients with multimorbidity [21], most current research focuses on comprehensive care programs for people with single diseases [22]. New emerging literature in primary care has put emphasis on two kinds of interventions that could inform optimal care: those based on a broad patient-centered care approach for patients with chronic diseases; and those specifically designed for patients with multimorbidity. Multimorbidity has been described above. Patient centered care is defined as a philosophy of care that encourages: a) shared control of the consultation decisions about interventions or management of the health problems of the patient, and/or b) a focus in the consultation on the patient as a whole person who has individual preferences situated within social contexts [23]. Effectiveness of those two kinds of interventions in primary healthcare have been documented in systematic reviews [20, 23, 24]. However the reviews did not report on specific elements associated with improved health-related outcomes in the patients; this represents an important gap in knowledge in addressing the needs of patients with multimorbidity [25].

The aim of this review is to identify the specific elements of patient-centered care and multimorbidity interventions that are associated with positive outcomes for patients. The findings will support a reorientation of care from a single disease focus to a multimorbidity focus and centering not only on disease but also on the patient and context.

## Method

In order to explore, map and synthesize the elements of patient-centered care interventions and interventions for patients with multimorbidity associated with positive health-related outcomes, a scoping review was conducted following the five stage approach suggested by Arksey and O’Malley’s (2005) [26]. A summary of the stages is described below.

### Stage 1: Identifying the research question

The research question for this scoping review was

- What are the elements of patient-centered care interventions and interventions for patients with multimorbidity associated with positive health-related outcomes for patients?

### Stage 2: Identifying relevant studies

To identify the relevant studies we reviewed four recent systematic reviews [9, 23, 24, 27], which to our knowledge were the only ones available on the topic that were published between 2012 and 2016. These systematic reviews formed the basis of our search for original studies because they included studies published between 1990 and 2015. By providing information on care programs and their impact on patients with multimorbidity (De Bruin), assessing the clinical impact of patient-centered care interventions (Dwamena) and evaluating effectiveness of interventions specifically designed for patients with multimorbidity (Smith 2012; 2016), these syntheses were aligned with the research question of our scoping review.

### Stage 3: Selecting studies

Two authors (MEP and MEM) independently assessed the eligibility of original studies. A three-step screening process was done: review of the abstract; review of the context, and review of the results. To be included in the mapping, studies had to be conducted in primary care and report at least one positive relationship with health-related outcomes.

As with all scoping reviews, the aim was to map the current literature on interventions with positive health-related outcomes, rather than to assess the quality of the particular studies chosen [26, 28]. Quality appraisal of included literature is not required when performing a scoping review [26, 29]. For this scoping review however, studies included had been assessed for quality to be included in their respective systematic reviews.

### Stage 4: Charting the data

Two authors (MEP and MEM) conducted comprehensive reading and inductive coding for each original study. The research team generated a template for data extraction using NVivo 10.

NVivo 10 software was used to classify studies, to extract the date and to create categories and sub-categories. Synthesis and interpretation of the extracted data were done for the following: 1) type of intervention; 2) elements of the intervention; and 3) health-related positive outcomes. For this scoping review the term “element” is used to define an “operational element of intervention” or the “process more likely to be related to the outcomes”. Elements may have been clearly identified by the authors of the original studies or deduced from the content of the studies included.

Data were classified by author, year of publication, study location, study population, context of care, study aim and methodology. Interventions, elements and health-related outcomes that emerged from the data analysis were then classified. Frequent meetings were held to compare and adjust the coding and reach agreement. The findings were then merged and coding was discussed between the two authors. As a final step, the findings were shared and agreed upon by the whole research team.

### Stage 5: Collating, summarizing and reporting the results

Co-interpretation and co-classification of types of intervention, elements of intervention and outcomes into common themes was performed by two authors (MEP and MEM.) They used the NVivo software to select and compile data and highlight the most frequently reported elements related to each intervention. Main interventions, their elements and outcomes were identified by consensus. The main interventions were classified according to a modified version of the Taxonomy of Interventions as reported by the Cochrane Effective Practice And Organisation of Care Review Group [30] used by Smith et al. (2012) (Table 1) [20].

**Table 1:** Modified version of Effective Practice and Organisation of Care Taxonomy

Categories	Examples
Patient-oriented interventions	For example, providing patient-oriented approaches, patient education or support for self management
Professional interventions	For example, training healthcare providers to improve their competencies and knowledge related to patients’ needs and patient-centered care
Organisational interventions	For example, supporting or providing case/care management decision process and evidence-based practice

## Results

### Search results

**Fig. 1:**
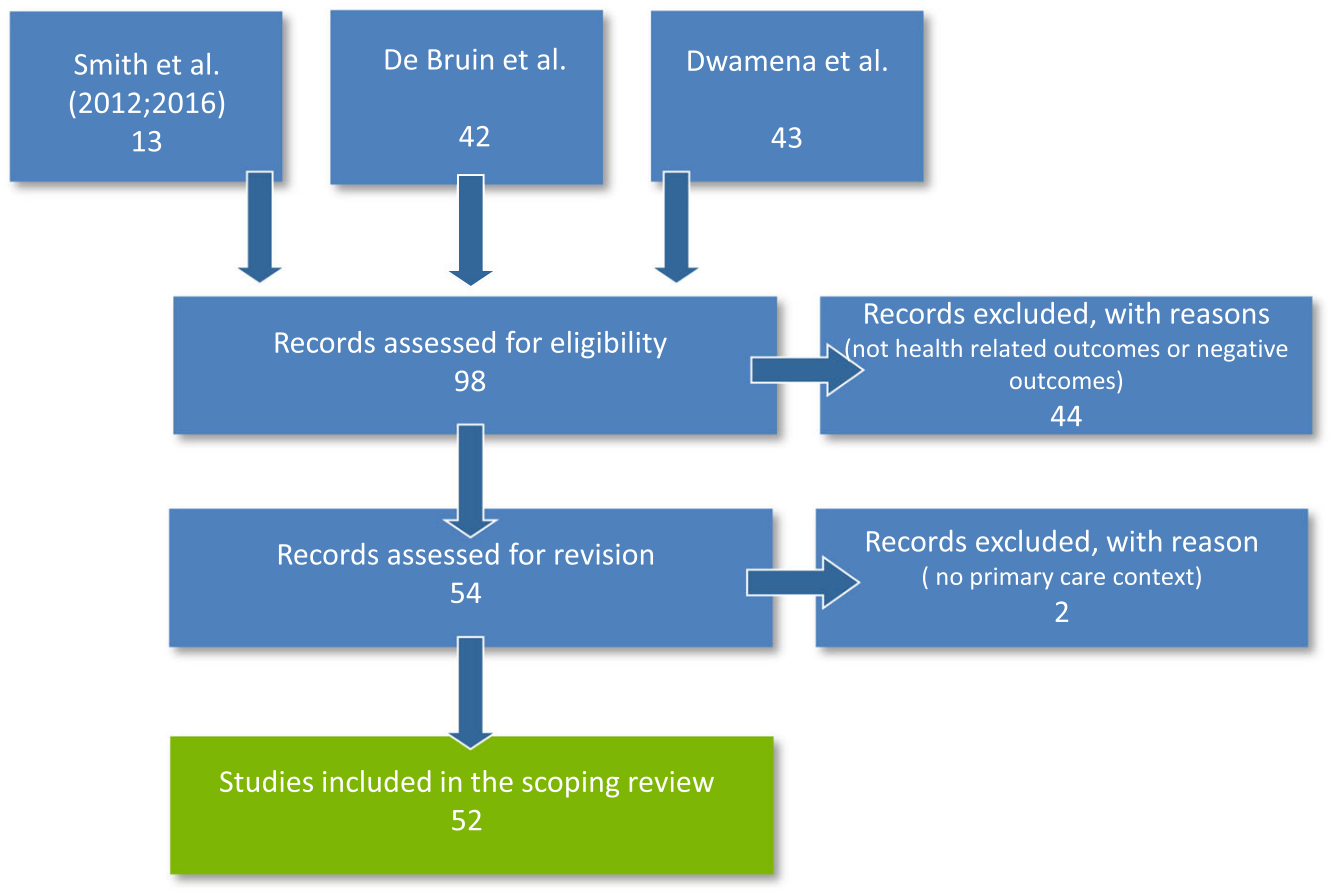
Flow diagram of the literature screening process

The four systematic reviews [20, 23, 24, 27] identified 98 original studies. Fifty-two were included in the scoping review. Figure 1 shows the detailed flow chart of the selection process of the studies included in this scoping review.

The studies included were published between 1995 and 2015, 85% of them after 2000. Appendix 1 (see Additional file 1) provides details of the studies included. The studies included for each initial systematic review are identified by asterisks i.e. a (De Bruin); b (Smith 2012); c (Dwamena 2012); and d (Smith 2016). No duplicates were found among studies. Studies used quantitative or qualitative designs and were mainly from the United States, followed by Canada, United Kingdom and the Netherlands. All studies were published in English. Patients of the included studies were adults, mostly 50 years and older.

### Interventions, elements of interventions and positive health-related outcomes

**Fig. 2:**
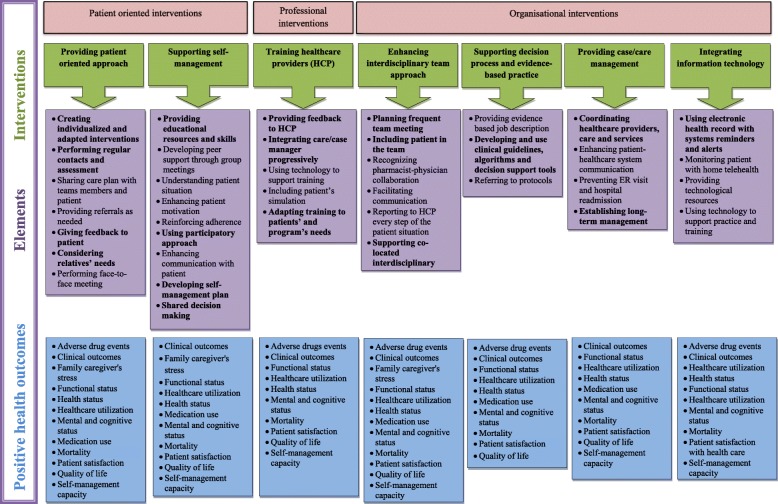
Interventions, elements and positive health-related outcomes

This scoping review identified seven types of interventions that could be further synthesized into three categories according to Smith et al. (2012;2016): patient-oriented interventions; professional interventions; and organisational interventions. The following section presents the three categories and seven types of interventions and their most important elements. Details and health-related outcomes linked to these elements are presented in Fig. 2.

### Patient-oriented interventions

#### Providing a patient-centered approach

Providing a patient-centered approach was one of the most important interventions in terms of positive health-related outcomes for patients with chronic diseases. A total of 37 studies used patient-centered interventions to enhance health-related outcomes for patients with chronic diseases [31–67]. Elements of the interventions were grouped into eight sub-categories. Of these sub-categories, three were more common: 1) Performing regular face-to face clinical evaluations and follow-up [31, 34–39, 42–48, 50–62, 64, 67]; 2) Creating individualized and adapted interventions [31, 36, 38, 41, 44, 47, 48, 51, 55, 57, 58, 62, 64, 65, 67]; and 3) Considering family or relatives needs [34, 37–39, 42–46, 49, 50, 52, 55, 56, 60, 62, 66]. Performing regular face-to-face clinical evaluations and follow-up was undoubtedly the element most frequently found in the literature. For example, Wright et al. (2007) used nurse case managers to follow low-income elderly patients with chronic conditions and functional impairment at high risk for rehospitalization or nursing home placement. The nurse case managers were required to call or visit patients as needed to ensure that care plans were implemented. They also accompanied patients to their healthcare visits if necessary [62]. Several studies also highlighted the importance of adapted and individualized interventions; an example being Dorr and al. (2006) who implemented a Chronic Care Model-based program including care managers located within multiplayer primary care clinics collaborating with physicians, patients, and other members of a primary care team to improve patient outcomes for a variety of conditions. One element of the intervention was the creation of an individualized care plan structured to reflect patient needs, specific conditions, personal challenges and goals [40]. Eakin et al. (2007) went one step further and included family and relatives needs’ in an individualized and culturally-adapted care plan in a program enhancing self-management with patients with multiple chronic diseases [44].

#### Supporting patient self-management

Self-management support interventions were included in 37 studies [25, 33, 35, 36, 38–48, 50–53, 55, 57, 58, 60, 61, 64–76]. The most frequently reported element was Providing educational resources and skills [40, 44–48, 51, 53, 57, 64, 65, 67, 69–72, 76]. This element was the basis for the majority of the self-management support interventions that could be done during individual or group meetings. For example, Ory et al. (2013) implemented a Chronic Disease Self-Management Program (CDSMP) among a national sample of patients with chronic diseases to improve patient health, healthcare services and healthcare utilization [72]. More specifically, this program supported patients by integrating small group workshops lead by peer leaders and face-to-face self-management meetings. Katon et al. (2010) preferred to use a 12-month individual follow-up with nurses in collaboration with primary care physicians [48]. The self-management intervention combined support for self-care with pharmacotherapy to control depression, hyperglycemia, hypertension, and hyperlipidemia [48]. Another example, not frequently found in literature, was a participatory approach to use empowerment strategies designed to promote positive attitudes, knowledge and skills to maintain and to enhance health, self-efficacy and patient participation. This approach with patients seemed to be a relevant and innovative element to consider. [53]

### Professional interventions

#### Developing training for healthcare providers

Supporting healthcare providers (HCP) through appropriate training was one of the most popular interventions in the literature associated with positive health-related outcomes. This scoping review found 34 studies reporting this intervention as an effective approach for people with multimorbidity [31–33, 36, 40, 41, 43, 46, 48, 52–55, 57, 58, 60, 62–69, 73–75, 77–83]. Adapting training to patient and program needs was the most frequent element found in 10 studies [32, 33, 36, 40, 41, 43, 58, 62, 64, 65, 77, 82]. For example, many studies in this category included comprehensive training for the healthcare professional to act as care manager [41, 58, 68]. Trainers providing feedback to HCP was also relevant to maximising impact on the patient health. [46, 48, 53, 64, 78, 80]. Among the six studies referring to this element, Gitlin (2006) described a process where the provider submitted taped treatment sessions to the investigators for review, received feedback and improved the intervention. The use of technology was also mentioned by others [32, 78, 80, 82]. Finally, three studies reported that the progressive integration of case/care manager in the clinical setting [32, 33, 40, 41, 53, 77] was having an impact on patient health-related outcomes and was appreciated by healthcare providers. For example, Boyd (2008) highlighted the success of involving the guided care nurse in a 3-month integration process into the practice through working with two physicians. This progressive integration into the work flow of the practice allowed to resolve problems and was essential to developing effective teamwork.

### Organisational interventions

#### Enhancing interdisciplinary team approach

Enhancing interdisciplinary team approach was the object of 24 studies [25, 34, 38–43, 48, 50, 51, 53–56, 58, 59, 61–64, 67, 74, 76]. Some of them highlighted the relevance of performing frequent team meetings [37, 43, 50, 51, 55, 58, 59, 76]. One particular aspect was including the patient in the team [50, 54, 56]. Patients were then involved in the different steps of their care. By being part of the process, their expectations were considered and the providers were able to personalize and maximize their approach. Four studies reported on the use of co-located teams and stressed the importance of supporting them [34, 37, 40, 43]. A typical organisation was an interdisciplinary team clinic including several healthcare professionals working together for a common group of patients.

#### Supporting the decision-making process and evidence-based practice

Supporting the decision-making process and evidence-based practice were included in 19 studies [32, 40, 41, 44, 47, 48, 54–56, 59, 60, 62–64, 69, 71, 75, 78, 79]. The development, the integration and the use of clinical guidelines, algorithms and decision support tools were the most frequently cited element [32, 40, 41, 44, 47, 48, 54–56, 59, 60, 62–64, 69, 71, 78, 79, 81]. For example, in a study to evaluate the efficacy of a nurse-care management system designed to improve outcomes in patients with complicated cases of diabetes, Taylor et al. (2003) specified that nurse-care managers used treatment algorithms developed by the Kaiser Permanente Medical panels based on national guidelines to titrate patient medications for diabetes, cholesterol, and hypertension [60]. Dorr et al. (2006), for their part, offered a clear evidence-based job description to facilitate the care manager’s integration and role development inside a co-located interdisciplinary team clinic for patients with multimorbidity. They also referred to protocols to help healthcare providers plan and coordinate care for patients with chronic diseases.

#### Providing case/care management

Including case/care management interventions in programs for patients with multiple chronic diseases was an innovative aspect found in literature but not frequently. Nineteen studies presented case/care management elements in their intervention. They were all published after 2003 [32, 34, 35, 38, 39, 41–43, 49, 50, 53–57, 62, 64, 67, 76]. The most important element found was the coordination of healthcare providers, care and services [20, 32, 38, 39, 42, 43, 53, 55, 57, 62, 64, 67, 76]. Douglas et al. (2007) provided a rich example of an inclusive case/care management intervention [42]. Advanced practice nurses had to coordinate follow-up services for patients, facilitate communication among patient families and healthcare providers, and provide supportive services for family members after hospital discharge [42]. The timeline for the follow-up varied greatly among interventions. For some, follow-up was maintained indefinitely [34, 54]. For most of the studies, the follow-up ended when patient objectives were reached [35, 38, 39, 41–43, 49, 50, 53, 55–57, 62, 64, 68, 76].

#### Integrating information technology

The use of information technology was included in 13 studies [32, 35, 40, 41, 49, 52, 54, 56, 63, 66, 76, 81, 82]. Using electronic medical records with system reminders and alerts was the most recurrent element [40, 41, 52, 54, 56, 63]. For example, Dorr et al. (2006) found that a comprehensive and locally developed electronic health record system with reminders and notes available to all clinicians had a positive impact on patient health. Finally, the last element found was the use of home telehealth [35, 49, 52, 56, 76]. Liddy et al. (2008), found that tele-homecare units installed in patient homes by nurse practitioners and a pharmacist had a positive impact on patient health. Using instructions integrated into individualized care plans, the participants of this study, with the support of a nurse practitioner, learned to use the units and peripheral devices in order to enter daily physiological values that were sent to a secure internet application allowing access to HCP. The technology was described as user-friendly by caregivers and patients and found to be useful in reducing the number of office visits while improving the tracking of patient health.

## Discussion

### Theme 1 – Key results

Results show that providing patient-oriented approaches, self-management support interventions (e.g. Providing educational resources and skills) and developing training for healthcare providers (e.g. Integrating care/case manager progressively within care setting) were the most frequent categories of interventions identified in this review with a potential to result in positive impacts for patients with chronic diseases. Therefore, those interventions, must be encouraged to address the concerns of patients with multimorbidity about their care and health. Care/case manager implementation was also found to be effective but less so. The care/case manager could play several roles that include helping patients identify and reach their goals and patient advocacy in their relationship with other health professionals [84–87].

### Theme 2 – Complex interactions (real world)

Elements of patient-centered interventions and interventions for patients with multimorbidity could be grouped into seven types of interventions linked to positive health-related outcomes. Those elements were likely synergistic and their combination favoured their success. The number of elements varied between four and eight depending on the intervention. The most important thing to understand is that patient-oriented interventions, professional interventions and organisational interventions present complex interactions and it is impossible to isolate one element to link it to a successful outcome. A single element cannot be linked to the success of the whole intervention. This reflects reality in practice and the need for pragmatic interventions adapted to patients, professionals and organizations.

### Theme 3 – Where this review fits

This scoping review combined patient-centered interventions with interventions for patients with multimorbidity that were associated with positive health-related outcomes (see Fig. 2). This review adds to the work previously conducted by Dwamena et al. (2012) (patient-centered interventions), de Bruin et al. (2012) and Smith et al. (2012;2016) (interventions for patient with multimorbidity), by allowing a broader interpretation of the findings [9, 23, 24, 27]. The combination of those studies reflects the actual interest in creating innovative and successful patient-centered interventions for patients with multimorbidity. By presenting the likely elements of successful interventions (see Fig. 2), this review has the potential to inform further development of innovative patient-centered interventions for persons with multimorbidity.

### Strengths and limitations

This review used pragmatic and flexible methods to respond to the stated objectives. This inductive process is based on knowledge from qualitative and quantitative research and is strongly grounded in current concerns of healthcare providers in primary healthcare. Even if two of the authors of this scoping review are among the leading authors worldwide on the topics of patient-centered care (MS) and multimorbidity (MF) only one of the studies included in the systematic reviews that constitute the basis of this work were conducted by one of the authors [81]. The presence of many authors and countries support the relevance of this topic.

Despite the strengths of this scoping review, certain limitations should be mentioned. The reader must be aware that all the included studies come from countries with strong primary healthcare systems and various contexts within these systems. Generalizing to other systems should be made with caution. We included only studies with positive patient’s related health outcomes to inform quickly the reader about: “what is the most effective?”. We were only interested in the positive effects in studies listed by Dwamena et al. (2012), de Bruin et al. (2012) and Smith et al. (2012;2016) and therefore only reported elements associated with positive effects. This is by no mean a guarantee that those elements would be associated with success if applied in a different context or environment. Including studies with negative findings may have counterbalanced the findings. From the studies included it was not possible to establish a clear relationship between individual elements and specific positive health-related outcomes as each study included several elements. In other words, the actual work does not permit us to isolate which specific elements of an intervention are the most effective because, in our view, it was the sum of elements included in the intervention that ensured positive effects. While some may call this a limitation, it reflects the reality of complex healthcare interventions.

## Conclusion

This scoping review was motivated by the need to identify and implement innovative patient-centered interventions adapted to patients with multimorbidity. There is an evidence-base for innovations to ensure patient-centered care for persons with multimorbidity. The unique contribution of this work is in integrating literature related to interventions on patient centered care and care for patients with multimorbidity. For future research, this scoping review is a starting point and there is a need to conduct other research, such as systematic review or to summarize other relevant literature such as policy documents, grey literature and unpublished papers, to put forward innovative ways to include patients with multimorbidity in primary healthcare services. At this time, with this scoping review, any single outcome could be related to more than one intervention and element.

Clinicians must consider every component of patient-centered-care that will engage patients. Policy makers should review the evidence before implementing any new intervention in primary care and be sure to plan for evaluation whenever they implement a new intervention so that we can collectively learn from these experiences in various contexts. Further research is needed to explore which specific elements were related to which specific health-related outcomes. Finally, further research should include the innovative but less frequently reported elements found in interventions, such as establishing a long-term management plan between patients with chronic disease and care/case managers, supporting co-located interdisciplinary teams, engaging the patient as a partner and using telehealth technologies to improve health-related outcomes.

## Additional file

Additional file 1: Synthesis Table of included studies. (DOCX 60 kb)

## Abbreviation

HCP: Healthcare providers
