# Supplementary material for: What are the effective elements in patient-centered and multimorbidity care? A scoping review
Source: BMC Health Serv Res. 2018 Jun 14;18:446. doi: 10.1186/s12913-018-3213-8 (PMC6001147; doi:10.1186/s12913-018-3213-8)
Supplement: Supplementary file 1 — Synthesis Table of included studies. (DOCX 60 kb) [file 12913_2018_3213_MOESM1_ESM.docx]

Additional file 1: Synthesis Table of included studies (n=52)

|  | **AUTHOR (YEAR)** | **COUNTRY** | **STUDY TYPE / POPULATION /**  **CONTEXT /** | **INTERVENTION TYPE** | **ELEMENTS OF THE INTERVENTION** | **HEALTH-RELATED POSITIVE OUTCOMES** |
| --- | --- | --- | --- | --- | --- | --- |
| 1 | Alamo (2002)^c^ | Spain | RCT (n=110)  Primary care patients with benign chronic musculoskeletal pain and fibromyalgia | Patient-oriented interventions   - Providing patient-oriented approach   Professional intervention   - Training healthcare providers | - Creating individualized and adapted interventions - Performing regular face-to-face clinical contacts and assessments | Clinical outcomes (self-rated pain)  Mental and cognitive status (anxiety)  Functional status |
| 2 | Bogner (2008)^b^ | USA | RCT (n=64)  Patients ≥ 50 years old with hypertension and depression | Patient-oriented interventions   - Providing patient-oriented approach - Supporting self-management   Professional intervention   - Training healthcare providers   Organisational interventions   - Enhancing interdisciplinary team approach - Supporting decision process and evidence-based practice - Providing case/care management | - Creating individualized and adapted interventions - Performing regular face-to-face clinical contacts and assessments - Providing educational resources and skills - Adapting training to patient and program needs - Providing feedback to HCP - Integrating care/case manager progressively - Planning frequent team meetings - Developing and using clinical guidelines, algorithms and decision-support tools - Coordinating healthcare providers, care and services | Self-management capacity (adherence to antihypertensive and antidepressant medications)  Clinical outcomes (blood pressure control)  Mental and cognitive status (depression) |
| 3 | Boult (2008)^a^ | USA | RCT (n=904)  Multimorbid people aged  ≥ 65 years with high probability of using healthcare services intensively/at  high risk for incurring high healthcare costs in the coming year. | Patient-oriented interventions   - Providing patient-oriented approach   Professional interventions   - Training healthcare providers   Organisation interventions   - Supporting decision process and evidence-based practice - Providing case/care management - Integrating information technology | - Performing regular face-to-face clinical contacts and assessments - Adapting training to patient and program needs - Integrating care/case manager progressively - Developing and using clinical guidelines, algorithms and decision-support tools - Coordinating healthcare providers, care and services - Using technology to support practice and training | Self-management capacity (goal setting)  Patient satisfaction |
| 4 | Boyd (2008)^a^ | USA | Nonrandomized prospective CT (n=150)  Multimorbid people aged  ≥65 years with high  probability of using healthcare  services intensively/at  high risk for incurring high  healthcare costs in the coming year. | Patient-oriented interventions   - Providing patient-oriented approach - Supporting self-management   Professional intervention   - Training healthcare providers | - Performing regular face-to-face clinical contacts and assessments - Providing educational resources and skills - Reinforcing adherence - Integrating care/case manager - Adapting training to patient and program needs - Integrating care/case manager progressively | Patient satisfaction with health care (physician–patient communication) |
| 5 | Boyd (2010)^a^ | USA | RCT (n=904)  Multimorbid people aged  ≥65 years with high  probability of using healthcare  services intensively/at  high risk for incurring high  healthcare costs in the coming year. | Patient-oriented interventions   - Providing patient-oriented approach - Supporting self-management   Professional intervention   - Training healthcare providers | - Performing regular contacts and assessments - Adapting training to patient and program needs - Integrating care/case manager progressively | Patient satisfaction with health care |
| 6 | Brown (2001)^c^ | Australia | RCT (N=318)  Patients with heterogeneous cancers, seeing one of 5 medical and 4 radiation oncologists for the first time | Professional intervention   - Training healthcare providers   Organisational intervention   - Support decision process and evidence-based practice | - Using technology to support training - Providing feedback to HCP - Developing and using clinical guidelines, algorithms and decision support tools | Mental and cognitive status (anxiety) |
| 7 | Burns (1995)^a^ | USA | RCT (N = 130)  ≥ 65-year-old hospitalized veterans with impairment of daily living activities, chronic disease, polypharmacy, or two or more hospitalizations in the previous year | Patient-oriented interventions   - Providing patient-oriented approach   Organisational interventions   - Enhancing interdisciplinary team approach - Providing case/care management | - Performing regular face-to-face clinical contacts and assessments - Supporting co-located interdisciplinary teams - Establishing long-term management | Health status  Medication use (reduction)  Mental and cognitive status (depression)  Quality of life (life satisfaction)  Mortality |
| 8 | Chassany (2006)^c^ | France | Randomized multicenter study (N=24)  ≥ 49-year-old patients with osteoarthritis and pain | Professional intervention   - Training healthcare providers   Organisational interventions   - Supporting decision process and evidence-based practice - Integrating information technology | - Providing feedback to HCP - Developing and using clinical guidelines, algorithms and decision support tools - Using technology to support practice and training | Clinical outcomes (pain)  Functional status |
| 9 | Chumbler (2004)^a^ | USA | Case control design (n=226)  Patients with primary diagnoses of hypertension, diabetes, heart or respiratory disease | Patient-oriented interventions   - Providing patient-oriented approach - Supporting self-management   Organisational interventions   - Providing case/care management - Integrating information technology | - Performing regular face-to-face clinical contacts and assessment - Providing educational resources and skills - Preventing emergency room visits and hospital readmissions - Providing patient-healthcare system communication - Monitoring patient with home telehealth | Functional status  Mental and cognitive status |
| 10 | Coleman (1999)^a^ | USA | Randomized controlled trial with 24-month follow-up.  (N=169)  ≥ 65 years old in primary care clinics with the highest risk for being hospitalized or experiencing functional decline. | Patient-oriented interventions   - Providing patient-oriented approach - Supporting self-management   Professional intervention   - Training healthcare providers   Organisational intervention   - Enhancing interdisciplinary team approach | - Creating individualized and adapted interventions - Performing regular clinical contacts and assessments - Providing educational resources and skills - Sharing care plan with team members and patient - Patient session with pharmacist - Developing self-management plan - Adapting training to patient and program needs - Recognizing pharmacist-physician collaboration | Health status  Functional status  Mental and cognitive status (depression)  Patient satisfaction |
| 11 | Counsell (2007)^a^ | USA | Controlled clinical trial (N = 951)  ≥ 65-year-old primary care patients with low income | Patient-oriented interventions   - Providing patient-oriented approach   Organisational interventions   - Enhancing Interdisciplinary team approach | - Performing regular face-to-face clinical contacts and assessments - Considering relatives' needs - Planning frequent team meetings - Supporting co-located interdisciplinary teams | Health status  Functional status (vitality)  Quality of life (social functioning)  Mental and cognitive status |
| 12 | Courtney (2009)^a^ | Australia | RCT (N = 128)    ≥ 65 years old with acute medical admission and at risk of readmission | Patient-oriented interventions   - Providing patient-oriented approach - Supporting self-management   Organisational interventions   - Providing case/care management | - Performing regular face-to-face clinical contacts and assessments - Creating individualized and adapted interventions - Considering relatives' needs - Providing educational resources and skills - Coordinating healthcare providers, care and services | Healthcare utilisation (Decrease in readmissions) |
| 13 | Coventry (2015)^d^ | UK | RCT (N=387)  Patients with depression and diabetes and/or heart disease from 36 UK general practices  Mean age 59  62% female | Patient-oriented interventions   - Providing patient-oriented approach - Supporting self-management   Professional intervention   - Training healthcare providers   Organisational interventions   - Enhancing interdisciplinary team approach - Providing case/care management | - Creating individualized and adapted interventions - Coordinating healthcare providers, care and services - Providing educational resources and skills - Adapting training to patient and program needs - Planning frequent team meetings - Coordinating healthcare providers, care and services | Mental and cognitive status (depression)  Patient satisfaction (perception of care, patient centeredness)  Self-management capacity |
| 14-15 | Daly (2005)^a^  Douglas (2007)^a^ | USA | RCT (Daly) and Prospective experimental design (Douglas) (N=334)  Adults chronically critically ill who had received 3 days of mechanical ventilation | Patient-oriented interventions   - Providing patient-oriented approach - Supporting self-management   Organisational interventions   - Enhancing interdisciplinary team approach - Providing case/care management | - Performing regular face-to-face clinical contacts and assessments - Considering relatives' needs - Providing educational resources and skills - Coordinating healthcare providers, care and services - Facilitating communication - Enhancing patient-healthcare system communication | Healthcare utilisation (reduction of re-hospitalisations)  Quality of life |
| 16 | Dijkstra (2006)^c^ | Netherlands | RCT (N=1350)  Patients with diabetes (Type 1 and Type 2) | Patient-oriented interventions   - Supporting self-management   Professional intervention   - Training healthcare providers   Organisational interventions   - Supporting decision process and evidence-based practice | - Providing educational resources and skills - Providing feedback to HCP - Developing and using clinical guidelines, algorithms and decision support tools | Clinical outcomes (diabetes) |
| 17-18 | Dorr (2006)^a^ Dorr (2008)^a^ | USA | RCT (N=3432)  Patients ≥ 65 years old with various and complex illnesses from 7 primary care clinics | Patient-oriented interventions   - Providing patient-oriented approach - Supporting self-management   Professional intervention   - Training healthcare providers   Organisational interventions   - Enhancing interdisciplinary team approach - Supporting decision process and evidence-based practice - Providing case/care management - Integrating information technology | - Creating individualized and adapted interventions - Providing educational resources and skills - Enhancing patient motivation - Integrating care/case manager progressively - Adapting training to patient and program needs - Facilitating communication - Supporting co-located interdisciplinary teams - Providing evidence-based job descriptions - Developing and using clinical guidelines, algorithms and decision support tools - Referring to protocols - Using electronic health records with system reminders and alerts - Providing technological resources | Clinical outcomes (HbA1C)  Mortality (for patients with diabetes mellitus)  Healthcare utilisation (decrease in hospitalisations) |
| 19 | Duke (2005)^a^ | USA | Pretest, post-test design (N=107)  ≥ 65 year-old frail community-dwelling patients | Patient-oriented interventions   - Providing patient-oriented approach - Supporting self-management   Professional intervention   - Training healthcare providers   Organisational interventions   - Enhancing interdisciplinary team approach - Providing case/care management | - Performing regular face-to-face clinical contacts and assessments - Considering relatives' needs - Adapting training to patient and program needs - Providing educational resources and skills - Planning frequent team meetings - Supporting co-located interdisciplinary teams - Coordinating healthcare providers, care and services | Decrease in halthcare utilisation |
| 20 | Eakin (2007)^b^ | USA | RCT (N=175)  200 Urban low income Latinos with multiple chronic condition | Patient-oriented interventions   - Providing patient-oriented approach - Supporting self-management   Organisational interventions   - Supporting decision process and evidence-based practice | - Performing regular face-to-face clinical contacts and assessments - Providing feedback to patients - Creating individualized and adapted interventions - Considering relatives' needs' - Providing educational resources and skills - Shared decision-making - Developing and using clinical guidelines, algorithms and decision support tools | Self-management capacity (health behaviors) |
| 21 | Garvey (2015)^d^ | USA | RCT (n=50)  Patients with multimorbidity (median 4.5 conditions)  Median age 66  64% female | Patient-oriented interventions   - Supporting self-management   Organisational interventions   - Enhancing interdisciplinary team approach - Providing case/care management | - Providing educational resources and skills - Coordinating healthcare providers care and services - Planning frequent team meetings - Peer support through group meetings | Patient satisfaction  Self-management capacity (self-efficacy, autonomy in daily activities and  quality of life, goal achievement) |
| 22-23 | Gitlin (2006)^b^  Gitlin (2009)^b^ | USA | Prospective randomized trial (N=319)  Community-living older people with functional difficulties | Patient-oriented interventions   - Providing patient-oriented approach - Supporting self-management   Professional intervention   - Training healthcare providers | - Performing regular face-to-face clinical contacts and assessments - Providing feedback to patients - Considering relatives' needs - Providing educational resources and skills - Shared decision-making - Providing feedback to HCP | Functional status (bathing, toileting, falls, home hazard,)  Self-management capacity (use of adaptive strategies)  Mortality |
| 24 | Hochalter (2010)^b^ | USA | RCT (n=79)  79 older adults with multiple chronic illnesses | Patient-oriented interventions   - Providing patient-oriented approach - Supporting self-management   Organisational interventions   - Supporting decision process and evidence-based practice | - Creating individualized and adapted interventions - Performing regular face-to-face clinical contacts and assessments - Providing educational resources and skills - Shared decision-making - Developing and using clinical guidelines, algorithms and decision support tools | Self-management capacity |
| 25 | Katon (2010)^b^ | USA | RCT (n=214)  Patients from 14 primary care clinics with poorly controlled diabetes, coronary heart disease, or both and coexisting depression | Patient-oriented interventions   - Providing patient-oriented approach - Supporting self-management   Professional intervention   - Training healthcare providers   Organisational interventions   - Enhancing interdisciplinary team approach - Supporting decision process and evidence-based practice | - Performing regular face-to-face clinical contacts and assessment - Creating individualized and adapted interventions - Providing educational resources and skills - Enhancing patient motivation - Providing feedback to HCP - Developing and using clinical guidelines, algorithms and decision support tools | Patient satisfaction  Clinical outcomes  Functional status  Health status |
| 26 | Kinmonth (1998)^c^ | UK | RCT (n=250)  Newly diagnosed diabetes type 2 patients from 41 primary care practices | Professional intervention   - Training healthcare providers   Organisational interventions   - Supporting decision process and evidence-based practice | - Adapting training to patient and program needs - Developing and using clinical guidelines, algorithms and decision support tools | Health status ( wellbeing)  Patient satisfaction (treatment) |
| 27 | Kobb (2003)^a^ | USA | Prospective,  quasi-experimental design (n=1401)  Veterans with high-cost medical care needs and high healthcare use in the year preceding enrollment | Patient-oriented interventions   - Providing patient-oriented approach   Organisational interventions   - Providing case/care management - Integrating information technology | - Considering relatives' needs - Enhancing patient-healthcare system communication - Monitoring patient with home telehealth | Patient satisfaction  Heathcare utilisation  Health status |
| 28 | Krause (2006)^a^ | USA | Pretest, post-test  design (n=39)  Patients with multiple chronic conditions | Patient-oriented interventions   - Providing patient-oriented approach - Supporting self-management   Organisational interventions   - Enhancing interdisciplinary team approach - Providing case/care management | - Performing regular face-to-face clinical contacts and assessments - Considering relatives' needs - Providing educational resources and skills - Planning frequent team meetings - Including patient in the team - Coordinating healthcare providers, care and services | Functional status (physical functioning)  Health status  Self-management capacity (personal control, health habits, diet, exercise)  Patient satisfaction |
| 29 | Léveillé (1998)^a^ | USA | RCT (N = 201)  ≥ 70 year-old chronically ill patients | Patient-oriented interventions   - Providing patient-oriented approach - Supporting self-management   Organisational interventions   - Enhancing interdisciplinary team approach | - Performing regular face-to-face clinical contacts and assessments - Providing referrals as needed - Providing referrals based on patients’ needed - Creating individualized and adapted interventions - Providing educational resources and skills - Enhancing patient motivation - Planning frequent team meetings - Report to HCP every step of patient situations | Functional status (activities of daily living)  Medication use  Self-management capacity (health habits, diet, exercise) |
| 30 | Liddy (2008)^a^ | Canada | Post-test study (N = 22)  ≥ 50 years old with chronic illnesses and a life expectancy of more than 6 months | Patient-oriented interventions   - Providing patient-oriented approach - Supporting self-management   Professional intervention   - Training healthcare providers   Organisational interventions   - Integrating information technology | - Performing regular face-to-face clinical contacts and assessments - Creating individualized and adapted interventions - Considering relatives' needs - Providing educational resources and skills - Providing feedback to HCP - Using electronic health records with system reminders and alerts - Monitoring patient with home telehealth | Patient satisfaction (quality of care) |
| 31 | Lorig (1999)^b^ | USA | RCT ( N= 952)  ≥ 40 -ld patients with a diagnosis of heart disease, lung disease, arthritis or stroke | Patient-oriented interventions   - Supporting self-management   Organisational interventions   - Supporting decision process and evidence-based practice | - Providing educational resources and skills - Shared decision-making - Developing and using clinical guidelines, algorithms and decision support tools | Mental and cognitive status  Patient satisfaction (communication with physicians)  Health status (self-reported health)  Functional status (less social activities limitations, disability, health distress and fatigue)  Healthcare utilisation (hospitalizations)  Self-management capacity |
| 32 | Markle-Reid (2006)^a^ | Canada | RCT (N=288)  ≥ 75 year-old frail people eligible for home care services | Patient-oriented interventions   - Providing patient-oriented approach - Supporting self-management   Professional intervention   - Training healthcare providers   Organisational interventions   - Enhancing interdisciplinary team approaches - Providing case/care management | - Performing regular face-to-face clinical contacts and assessment - Providing educational resources and skills - Using participatory approach - Providing feedback to HCP - Integrating care/case manager progressively - Facilitating communication - Coordinating healthcare providers, care and services | Quality of life (defined by mental health, depression and perceived social support) |
| 33 | Martin (2015)^d^ | Australia | RCT (N = 66)  Patients with headache and depression | Patient-oriented interventions   - Supporting self-management   Professional intervention   - Training healthcare providers   Organisational interventions   - Supporting decision process and evidence based-practice - Integrating information technology | - Providing educational resources and skills - Adapting training to patient and program needs - Developing and using clinical guidelines, algorithms and decision support tools. - Providing technological resources. | Clinical outcomes (headaches)  Mental and cognitive status (depression) |
| 34 | Mitton (2007)^a^ | Canada | Pretest, ost-test  design (N = 37)  Older patients with high healthcare needs and complex or chronic medical problems | Patient-oriented interventions   - Providing patient-oriented approach - Supporting self-management   Professional interventions   - Training healthcare providers   Organisational interventions   - Enhancing interdisciplinary team approach - Supporting decision process and evidence-based practice - Providing case/care management - Integrating information technology | - Performing regular face-to-face clinical contacts and assessments - Providing educational resources and skills - Creating individualized and adapted interventions - Including patient in the team - Developing and using clinical guidelines, algorithms and decision support tools - Establish long-term management - Using electronic health records with systems reminder and alerts | Patient satisfaction  Mental and cognitive status (anxiety)  Healthcare utilization |
| 35 | Morgan (2013)^d^ | Australia | RCT (N= 400)  Patients with depression and diabetes and/or heart disease in 11 general practices | Patient-oriented interventions   - Providing patient-oriented approach - Supporting self-management   Professional intervention   - Training healthcare providers   Organisational interventions   - Enhancing interdisciplinary team approach - Providing case/care management | - Performing regular face-to-face clinical contacts and assessment - Creating individualized and adapted interventions - Sharing care plan with team members and patient - Providing educational resources and skills - Adapting training to patient and program needs - Planning frequent meetings - Coordinating healthcare providers, care and services | Mental and cognitive status (depression)  Clinical outcomes (HbA1C, Blood pressure, cholesterol, mean BM )  Medication use  Self-management capacity (smoking, alcohol, exercise,) |
| 36 | Naylor (2004)^a^ | USA | RCT (N = 239)  ≥ 65 year-old patients hospitalized with heart failure | Patient-oriented interventions   - Providing patient-oriented approach - Supporting self-management   Professional intervention   - Training healthcare providers   Organisational interventions   - Enhancing interdisciplinary team approach - Supporting decision process and evidence-based practice - Providing case/care management | - Performing regular face-to-face clinical contacts and assessments - Creating individualized and adapted interventions - Considering relatives' needs - Providing educational resources and skills - Planning frequent team meetings - Developing and using clinical guidelines, algorithms and decision support tools - Coordinating healthcare providers, care and services | Healthcare utilization  Quality of life (physical dimension)  Patient satisfaction |
| 37 | Ory (2013)^b^ | USA | Pre-post longitudinal design (N= 1170)  Adults from a chronic disease self-management program | Patient-oriented interventions   - Supporting self-management | - Providing educational resources and skills - Enhancing communication with patient | Patient satisfaction (health care experience)  Self-management capacity (self-care behaviors, medication adherence, literacy)  Health care utilisation (emergency room visits, hospitalization) |
| 38 | Procter (2006)^a^ | UK | Pretest, post-test design (N = 25)  ≥ 65 year-old people with a diagnosis of heart failure or COPD and early stage dementia, living at home, and with multiple hospital admissions | Patient-oriented interventions   - Providing patient-oriented approach   Organisational interventions   - Enhancing interdisciplinary team approach - Supporting decision process and evidence-based practice - Providing case/care management - Integrating information technology | - Performing regular face-to-face clinical contacts and assessments - Considering relatives' needs - Including patient in the team - Developing and using clinical guidelines, algorithms and decision support tools - Using electronic health records with system reminders and alerts - Monitoring patient with home telehealth | Patient satisfaction (communication with primary care team)  Healthcare utilization |
| 39 | Rose (2008)^a^ | USA | Pretest, post-test design (N = 175)  Urban African American adults with low income and ≥ 1 chronic condition | Patient-oriented interventions   - Supporting self-management   Professional intervention   - Training healthcare providers | - Peer support through group meetings - Adapting training to patient and program needs | Self-management capacity (health behavior)  Health status  Patient satisfaction (communication) |
| 40 | Roter (1995)^c^ | USA | RCT (N= 648)  Patients in primary care | Professional intervention   - Training healthcare providers   Organisational intervention   - Integrating information technology | - Providing feedback to HCP - Using technology to support practice and training | Mental and cognitive status (emotional distress) |
| 41 | Smith (2006)^c^ | USA | Clinical trial (N=206)  Patients 18 to 65 years old with 2 consecutive years of high utilization | Patient-oriented interventions   - Providing patient-oriented approach - Supporting self-management   Professional intervention   - Training healthcare providers   Organisational interventions   - Providing case/care management | - Performing regular face-to-face clinical contacts and assessment - Creating individualized and adapted interventions - Understanding patient situation - Providing educational resources and skills - Enhancing patient motivation - Coordinating healthcare providers, care and services | Mental and cognitive status |
| 42 | Sommers (2000)^b^ | USA | Concurrent controlled cohort study (N= 543)  Chronically ill patients from 18 private primary care practices | Patient-oriented interventions   - Providing patient-oriented approach - Supporting self-management   Professional intervention   - Training healthcare providers   Organisational interventions   - Enhancing interdisciplinary team approach | - Performing regular face-to-face clinical contacts and assessments - Shared decision-making - Creating individualized and adapted interventions - Adapting training to patient and program needs - Planning frequent team meetings | Healthcare utilization  Health status |
| 43 | Song (2005)^c^ |  | RCT (N= 32)  Patients undergoing cardiac surgery and their surrogates | Patient-oriented interventions   - Providing patient-oriented approach - Supporting self-management   Professional intervention   - Training healthcare providers | - Creating individualized and adapted interventions - Providing educational resources and skills - Adapting training to patient and program needs | Mental and cognitive status (anxiety) |
| 44 | Sorlie (2007)^c^ | Norway | Randomized trial (N=109)  Patients following coronary artery bypass surgery | Patient-oriented interventions   - Providing patient-oriented approach - Supporting self-management   Professional intervention   - Training healthcare providers   Organisational interventions   - Integrating information technology | - Considering relatives' needs - Providing education resources and skills - Adapting training to patient and program needs - Providing technological resources | Mental and cognitive status (anxiety and depression) |
| 45 | Sorrento (2007)^a^ | USA | Pretest, post-test design (N = 273)  ≥ 75 year-old frail, at-risk, medically complex and community-dwelling adults | Patient-oriented interventions   - Providing patient-oriented approach   Organisational interventions   - Enhancing interdisciplinary team approach - Supporting decision process and evidence-based practice | - Performing regular face-to-face clinical contacts and assessments - Planning frequent team meetings - Developing and using clinical guidelines, algorithms and decision support tools - Recognizing pharmacist-physician team | Medication use (problems) |
| 46 | Stewart (2007)^c^ | Canada | RCT (N= 102)  Patients with breast cancer | Professional intervention   - Training healthcare providers   Organisational interventions   - Supporting decision process and evidence-based practice - Integrating information technology | - Providing feedback to HCP - Developing and using clinical guidelines, algorithms and decision support tools - Using technology to support practice and training | Patient satisfaction |
| 47 | Taylor (2003)^a^ | USA | RCT (N = 169)  Patients with longstanding diabetes, and ≥ 1 major medical comorbid conditions, and HbA ˃ 10% | Patient-oriented interventions   - Providing patient-oriented approach - Supporting self-management   Professional intervention   - Training healthcare providers   Organisational interventions   - Supporting decision process and evidence-based practice | - Performing regular face-to-face clinical contacts and assessment - Considering relatives' needs - Providing educational resources and skills - Developing self-management plan - Developing and using clinical guidelines, algorithms and decision-support tools | Clinical outcomes (HbA1c) |
| 48 | Tibaldi (2004)^a^ | Italy | RCT (N=109)  Patients with severe dementia requiring admission to the Emergency Department | Patient-oriented interventions   - Providing patient-oriented approach - Providing self-management   Organisational interventions   - Enhancing interdisciplinary team approach | - Performing regular face-to-face clinical contacts and assessment - Providing educational resources and skills - Planning frequent team meeting | Family caregiver's stress (reduced)  Cognitive and mental status (behavioral disturbances)  Medication use (antipsychotic) |
| 49 | Wakefield (2012)^d^ | USA | RCT (N=302)  Adults with hypertension and diabetes | Patient-oriented interventions   - Providing self-management   Organisational interventions   - Enhancing interdisciplinary team approach - Providing case/care management - Integrating information technology | - Providing educational resources and skills - Performing frequent team meeting - Coordinating healthcare providers, care and services - Monitoring patient with home telehealth. | Clinical outcomes (HbA1c and blood pressure)  Self-management capacity **(**Medication adherence, knowledge) |
| 50 | Wilkinson (2008)^c^ | UK | RCT (N=172)  Cancer / palliative care patients | Professional interventions   - Training healthcare providers   Organisational interventions   - Integrating information technology | - Adapting training to patient and program needs - Including patient’s simulation - Providing technological resources | Patient satisfaction |
| 51 | Wright (2007)^a^ | USA | Post-test only  study (without  control) (pilot)  (N = 118)  Older people with low income, chronic conditions, functional impairment and high risk for early hospitalization or nursing home placement | Patient-oriented interventions   - Providing patient-oriented approach   Professional interventions   - Training healthcare providers   Organisational interventions   - Enhancing interdisciplinary team approach - Supporting decision process and evidence-based practice - Providing case/care management | - Creating individualized and adapted interventions - Performing regular face-to-face clinical contacts and assessments - Considering relatives' needs - Adapting training to patient and program needs - Planning frequent team meetings - Developing and using clinical guidelines, algorithms and decision support tools - Referring to protocols - Coordinating healthcare providers, care and services | Clinical outcomes (Blood sugars)  Functional status |
| 52 | Zhang (2008)^a^ | USA | Pre and post quasi-experimental  design (N = 65132)  Medicaid patients with at least one of the following conditions: diabetes, hypertension/congestive heart failure, depression, gastro-esophageal reflux disease/peptic ulcer disease and Asthma/COPD and/or comorbidities | Patient-oriented interventions   - Providing patient-oriented approach   Professional intervention   - Training healthcare providers   Organisational interventions   - Enhancing interdisciplinary team approach - Supporting decision process and evidence-based practice - Integrating information technology | - Planning frequent team meetings - Developing and using clinical guidelines, algorithms and decision support tools - Using electronic health record with systems reminders and alerts - Recognizing pharmacist-physician collaboration | Healthcare utilization  Adverse drug events  Quality of life |
